# Circulating tumour cells and cell-free DNA as a prognostic factor in metastatic colorectal cancer: the OMITERC prospective study

**DOI:** 10.1038/s41416-021-01399-6

**Published:** 2021-05-05

**Authors:** Francesca Salvianti, Stefania Gelmini, Irene Mancini, Mario Pazzagli, Serena Pillozzi, Elisa Giommoni, Marco Brugia, Francesco Di Costanzo, Francesca Galardi, Francesca De Luca, Francesca Castiglione, Luca Messerini, Pamela Pinzani, Lorenzo Antonuzzo

**Affiliations:** 1 Department of Experimental and Clinical Biomedical Sciences “Mario Serio”, University of Florence, Florence, Italy; 2 Clinical Oncology Unit, Careggi University Hospital, Florence, Italy; 3 Sandro Pitigliani Medical Oncology Department, Hospital of Prato, Istituto Toscano Tumori, Prato, Italy; 4 Histopathology and Molecular Diagnostics Unit, Careggi University Hospital, Florence, Italy; 5 Department of Experimental and Clinical Medicine, University of Florence, Florence, Italy

**Keywords:** Prognostic markers, Colon cancer

## Abstract

**Background:**

Within the OMITERC prospective study (OMIcs application from solid to liquid biopsy for a personalised ThERapy of Cancer), we explored the prognostic role of liquid biopsy encompassing cell-free DNA (cfDNA) and circulating tumour cells (CTCs) in KRAS mutated metastatic colorectal cancer (mCRC).

**Methods:**

We defined a workflow including pre-analytical and analytical procedures collecting blood before therapy and every 3 months until disease progression (PD). CTCs were counted by CellSearch® and isolated by DEPArray™. NGS sequencing of CTCs and cfDNA was performed using a panel of cancer/CRC related genes respectively.

**Results:**

KRAS mutational status was mostly concordant between tumour tissues and liquid biopsy. The percentage of cfDNA samples with mutations in CRC driver genes was in line with literature. In longitudinal monitoring circulating biomarkers anticipated or overlapped conventional diagnostic tools in predicting PD. The presence of CTCs at baseline was confirmed a negative prognostic marker.

**Conclusions:**

Cell-free DNA and CTCs are readily available candidates for clinical application in mCRC. While CTCs demonstrated a prognostic significance at baseline, cfDNA was confirmed an easily accessible material for monitoring the mutational status of the tumour over time. Moreover, in the longitudinal study, the two markers emerged as complementary in assessing disease progression.

## Background

Precision medicine is an emerging approach for disease treatment and prevention that takes into account individual variability in genes, environment and lifestyle. Although this approach can be employed in several areas of medicine, precision medicine is most likely to introduce major changes in cancer treatment giving precise and individualised solutions to tailor specific therapy to patients, based on both their germline genetic profile (e.g. pharmacogenomics) and cancer somatic genomic variants.

OMITERC (OMIcs application from solid to liquid biopsy for a personalised ThERapy of Cancer) was a data-sharing project aimed to develop a ‘real world’ dataset linking cancer genomic and pharmacogenomics data with clinical outcomes from KRAS mutated metastatic colorectal cancer (mCRC) with the goal to identify a pool of molecular biomarkers useful for the stratification of patients in appropriate risk groups and optimisation of therapeutic regimens. Within OMITERC, our research team was the responsible of the WP dedicated to the liquid biopsy implemented by cell-free DNA (cfDNA) analysis, circulating tumour cell (CTC) identification and molecular characterisation. To this purpose, blood samples were collected at the time of enrolment and every 3 months after the recruitment, until disease progression.

Liquid biopsy allows non-invasive real-time monitoring of tumour evolution and therapeutic efficacy in cancer patients. Because of its clinical implications for personalised medicine, liquid biopsy has become the subject of a huge number of studies.^1^ In fact, the study of the liquid biopsy has been employed for the early detection of cancer, the identification of therapeutic targets and the monitoring of the response to therapy, as well as the study of mechanisms underlying the disease progression.^2^ Despite the impressive advancements, liquid biopsy has not been implemented yet into routine clinical practice in CRC. To this purpose, different biological and technological challenges need to be solved.

Here we report the results of the analysis performed by the adoption of integrated and standardised workflows of the liquid biopsy from 20 KRAS mutated mCRC patients before receiving first-line chemotherapy (CT). CTC counting by CellSearch® and cfDNA analysis by NGS were performed to identify a panel of somatic mutations with potential prognostic significance in KRAS mutated mCRC. Although both liquid biopsy methods are being increasingly proposed in various tumours including CRC, very limited comparative data are available between analysis of cfDNA and CTCs. With this study, we aimed to test their performance in terms of successful detection as biomarkers in patients with mCRC.

## Methods

### Patients

Twenty patients with mCRC treated at the Medical Oncology Unit of Careggi University Hospital, Florence, Italy, from April 2016 to October 2018, were enrolled. The study was approved by the Local Ethical Committee and written informed consent to participate was obtained from all patients. Among the inclusion criteria, there were the availability of a tumour tissue sample (primary tumour and/or metastatic sites), stage IV, RAS mutation positive (NRAS or KRAS) assessed on tumoural tissue specimen, at least one measurable lesion according to RECIST 1.1, adequate liver, renal and bone marrow function. Previous oxaliplatin-based adjuvant CT, previous treatment with anti-EGFR monoclonal antibodies, symptomatic grade >1 peripheral neuropathy, and any contraindications to study drugs were exclusion criteria. Adjuvant fluoropyrimidine monotherapy was allowed only if progressive disease (PD) occurred more than 6 months after the completion of adjuvant CT. According to the RECIST, PD is diagnosed under two conditions: an increase in size of pre-existing lesions and the appearance of new lesions. Patients’ characteristics are summarised in Supplementary Table S1. All blood samples were collected before treatment (time 0).

### Study design

Firstly, a standardisation of integrated workflows, from the pre-analytical sample handling procedures to the molecular analysis of liquid biopsy-derived samples, as well as data analysis and interpretation is strongly required.^3^ We dedicated particular attention to the pre-analytical phase of the project by adopting procedures in accordance to international standards [CEN/TS 16835-3:2015 (WI = 00140091) Molecular in vitro diagnostic examinations— Specifications for pre-examination processes for venous whole blood—Part 3: Isolated circulating cell-free DNA from plasma; ISO 20186-3, Molecular in vitro diagnostic examinations—Specifications for pre-examination processes for venous whole blood—Part 2: Isolated cell-free DNA]. We adopted certified blood collection tubes with different stabilisers specific for CTC detection and counting and for cfDNA analysis, having previously tested their suitability for the respective downstream application. We defined storage and transport conditions and adopted a standardised automated cfDNA isolation protocol, quantity and quality assessment of cfDNA.

For CTC counting, we used the CellSearch® system (Menarini Silicon Biosystems, Inc), being the only FDA approved CTC quantification technology. On a subset of samples with a CTC number of at least five cells, after enrichment by CellSearch®, CTCs were isolated by the DEPArray™ system to obtain single CTC samples for the analysis of the mutational status of multiple genes involved in cancer. For CTC molecular characterisation by NGS we applied a previously validated workflow^4,5^ using a panel of genes designed to investigate genomic ‘hot spot’ regions of 50 oncogenes and tumour suppressor genes. Cell-free DNA mutational status was assessed by NGS using a smaller gene panel strictly related to CRC and an NGS approach specific for cfDNA based on tag sequencing barcodes limiting false positive results.

### Blood collection

For each patient, peripheral blood was collected in a CellSave Preservative tube (CellSearch; Menarini Silicon Biosystems) for CTC enrichment and enumeration and in a cell-free DNA BCT (Streck) tube for cfDNA molecular analysis at Careggi University Hospital, Florence, Italy. On the same day of the collection blood samples were sent at room temperature to the laboratory. All the samples were then processed for CTC counting and plasma separation within 24 h after blood collection. Longitudinal blood samples were collected at 48 h, 40 days, 3 months and then every 3 months till disease progression.

### Cell enrichment and enumeration of CTCs

CTC enrichment and enumeration were performed by the CellSearch® System (Menarini Silicon Biosystems, Italy). 7.5 mL of whole blood were processed using the CellSearch® Epithelial Cell kit (Menarini Silicon Biosystems) which selects EpCAM positive cells, using ferrofluids particles coated with EpCAM antibody. Cells were stained with the nuclear dye 4′,6′-diamino-2-phenylindole (DAPI), anti-cytokeratin 8, 18 and 19-phycoerythrin (PE) labelled antibodies and anti-CD45 antibody labelled with allophycocyanin (APC). After enrichment, isolated and stained cells were resuspended in the MagNest Device (Menarini Silicon Biosystems); labelled cells were analysed in the CellTracks®Analyzer II (Menarini Silicon Biosystems) and then cells were identified and enumerated according to the criteria specified by the manufacturer’s instructions. Supplementary Fig. S1 reports some representative examples of CTCs from mCRC patients as they appear in the CellSearch® image gallery. CTCs show positive fluorescent signals for cytokeratins, no signal for CD45, and nucleus (DAPI signal) within cytoplasm.

### Single-cell analysis

Single-cell analysis was performed on samples with at least 5 CTCs per 7.5 ml plasma, according to the CellSearch® count. CTCs, enriched and enumerated by CellSearch®, were subsequently isolated by DEPArray™ to obtain single-cell samples to be submitted to whole genome amplification (WGA) using the Ampli1 WGA kit (Menarini Silicon Biosystems) for the assessment of the mutational status of multiple genes involved in cancer by NGS, using a panel designed to investigate genomic “hot spot” regions of 50 oncogenes and tumour suppressor genes. This optimised workflow for the molecular characterisation of multiple genes in single cells by NGS has been previously published.^4,5^

### Plasma separation, DNA extraction and quantification

Plasma was separated from blood collected in BCT with cell-free DNA BCT tubes according to the manufacturer’s instructions; a centrifugation step of 10 min at 1600 × *g* was followed by a second step at 16,000 for 10 min, at room temperature. Plasma aliquots were stored at −80 °C. DNA was extracted from 2 mL of plasma, using the QIAsymphony Circulating DNA Kit (Qiagen, Hilden, Germany) on the QIAsymphony instrument following the manufacturer’s instructions.

### Next generation sequencing (NGS)

Sequencing analysis was performed on the Ion S5 system (Thermofisher Scientific, USA). Libraries from single CTC WGA products were prepared using the Ion AmpliSeq™ Cancer Hotspot Panel v2 (Thermofisher Scientific) designed to target 207 amplicons covering mutations from 50 oncogenes and tumour suppressor genes as reported in Supplementary Table S2.

DNA quantification was assessed using the Qubit 3.0 Fluorometer (Thermofisher Scientific). Ten nanograms of DNA were used to prepare barcoded libraries using the Ion AmpliSeq™ Library kit 2.0 and Ion Xpress™ barcode adapters (Thermofisher Scientific). The libraries were purified with the Agentcourt AMPure XP reagent (Beckman Coulter) and quantified with the Ion Library Quantitation Kit (Thermofisher Scientific) on the StepOne Plus system (Thermofisher Scientific). Control libraries from single WBCs and gDNA from the same patients were prepared according to the same procedure.

Template preparation for sequencing was performed using the Ion 520™ & Ion 530™ Kit-OT2 (Thermofisher Scientific) on the Ion OneTouch™ 2 System and Ion One Touch ES. Sequencing was performed on the Ion S5 using Ion 520 Chips (Thermofisher Scientific). The run was set in order to achieve at least 1000× coverage for each sample (10 samples per chip).

Data analysis was carried out using the Ion Reporter Software 5.10 and the workflow AmpliSeq CHPv2 single sample. Variants common to single white blood cells and/or gDNA were excluded.

Libraries from cfDNA were prepared using the Oncomine™ Colon cfDNA Assay to detect colon (or other related gastrointestinal) tumour-derived DNA (ctDNA) in cfDNA. The panel allows the analysis of single nucleotide variants and short indels that are frequently detected in colon/gastrointestinal cancers. More than 240 hotspots in 14 genes are covered: AKT1, APC, BRAF, CTNNB1, EGFR, ERBB2, FBXW7, GNAS, KRAS, MAD4, MAP2K1, NRAS, PIK3CA, TP53. The method involves the use of tag sequencing technology. Libraries were checked by capillary electrophoresis on the Agilent Bioanalyzer using the Agilent High Sensitivity DNA kit and quantified by the Ion Library Quantitation Kit (Thermofisher Scientific) on the StepOne Plus system (Thermofisher Scientific). Template preparation for sequencing was performed using the Ion 520™ & Ion 530™ Kit-OT2 (Thermofisher Scientific) on the Ion OneTouch™ 2 System and Ion One Touch ES. Sequencing was performed on the Ion S5 using Ion 530 Chips (Thermofisher Scientific) analysing 6 samples per chip. Data analysis was carried out using the Ion Reporter Software 5.10 and the workflow Oncomine Colon Liquid Biopsy-w1.4-DNA-Single Sample. The uniformity of coverage (a quality parameter of sequencing) was 100% in all the analysed samples.

### Statistical analysis

Demographic and clinical data were analysed using descriptive statistics. Overall survival (OS) was defined as the time from the enrolment to death from any cause. Progression-free survival (PFS) was defined as the time from the enrolment to disease progression, relapse, or death from any cause. Statistical comparisons for categorical variables were performed using chi-square test or the Fisher’s exact test when appropriate. In order to define the differences in survival rate between positive and negative CTC patients, the univariate Cox’s model was adopted, including log-rank test and Kaplan–Meier (KM) curves. In particular, time-to-event endpoints were estimated using the KM method and survival distributions for specific subgroups of patients were tested with log-rank test. Hazard ratios (HR) and 95% confidence intervals (CI) were computed. A *p*-value of 0.05 or lower was considered to be statistically significant. Analyses were performed using the IBM SPSS Statistics software package, release 25.0.0.1 and survival analysis was performed using R (version 4.0.2, R Foundation for Statistical Computing, Vienna, Austria, https://www.R-project.org/) package ‘survival’; data were plotted with package ‘ggplot2’.

## Results

The average age of the mCRC patients enrolled in the OMITERC study was 65 years (range, 45**–**84 years; Supplementary Table S1A), 50% were males and 50% females. Twelve colon tumours (60%) were right-sided and 8 (40%%) left-sided. With regard to the number of metastatic sites, 6 (30%) patients had one site, 7 (35%) patients were found to have two sites and 7 (35%) three or more sites. Supplementary Table S1B reports the sites of metastases in CRC patients of our cohort. All patients were RAS mutated as assessed on tumour tissue specimen.

### CTC enumeration and correlation with outcome

All samples were successfully processed for CTC counting except for two basal blood samples (time 0) not assessable for CTC presence by CellSearch® due to blood coagulation (COL 10) and technical failure (COL 17). CTCs were detected in 7/18 (39 %) basal samples from mCRC patients with a median number of CTCs of 0 (range, 0–12) in the whole cohort and in positive samples of 3 (range, 1–12), with 4/7 (22%) samples with more than 3 CTCs/7.5 ml of blood at time 0. Considering the best response to first-line CT, the percentage of patients with PD was higher in patients positive for the presence of CTCs (Table 1).

**Table 1:** Different cut-offs of CTC counts determined by CellSearch® in liquid biopsy of mCRC patients at time 0 correlated to best response to first line CT.

CTC count at time 0	Best response to first-line CT		
	SD	PR	PD
≥1	2/7 (29%)	2/7 (29%)	3/7 (42%)
<1	3/11 (27%)	7/11 (64%)	1/11 (9%)
≥3	1/4 (25%)	2/4 (50%)	1/4 (25%)
<3	4/14 (29%)	8/14 (57%)	2/14 (14%)

*CT* chemotherapy, *SD* stable disease, *PR* partial response, *PD* progressive disease.

**Fig. 1:**
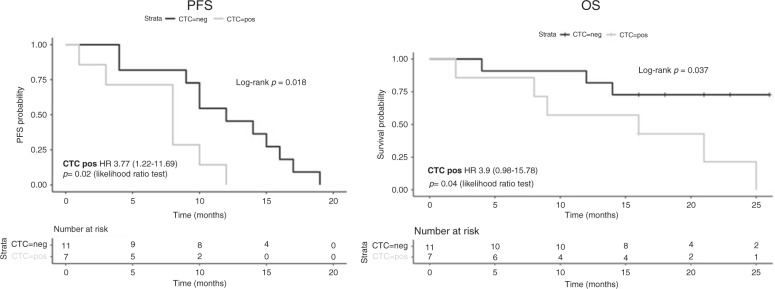
Kaplan–Meier estimates of probabilities of progression-free survival (PFS) and overall survival (OS) of mCRC patients presenting (CTC POS) or not (CTC NEG) CTCs at baseline (time 0). Time on the *x*-axis is expressed in months.

Cox analysis evidenced a HR of 3.7 (CI 1.218–11.69), *p* = 0.02 and 3.9 (CI 0.98–15.78), *p* = 0.04 for PFS and OS respectively. In addition, log-rank test showed that both PFS and OS in patients with peripheral blood CTCs were significantly lower than in patients without CTCs (*p* = 0.018 and p = 0.037 respectively), (Fig. 1).

### Molecular characterisation of the liquid biopsy

#### Single CTC sequencing

Only the two samples (COL 05 and COL 07) showing more than 5 CTCs according to the CellSearch® count could be selected for the molecular analysis of single CTCs. One CTC from patient COL 05 and four CTCs from patient COL 07 were sorted singularly by DEPArray™ and sequenced upon whole genome amplification as described in the ‘Methods’ section. Variants detected in CTCs are reported in Table 2.

**Table 2:** Sequence variants detected in single CTCs.

Patient	Sample	Genes	Exon	Amino acid change	Coding	COSMIC/ dbSNP
COL 05	WGA CTC1	FBXW7	9	p.L457fs	c.1368delC	
COL 07	WGA_CTC1	APC	16	p.Q1367*	c.4099 C>T	COSM13121
		CDH1	3	Synonymous	c.243 T>C	rs765879351
		KRAS	2	p.G12S	c.34 G>A	COSM517
	WGA_CTC2	APC	16	p.Q1367*	c.4099 C>T	COSM13121
		KRAS	2	p.G12S	c.34 G>A	COSM517
	WGA_CTC3	PDGFRA	18	Synonymous	c.2541 G>T	rs370355869
	WGA_CTC4	PTEN	8	Synonymous	c.858 C>A	

KRAS mutation was detected only in two CTCs from patient COL 07 and not in the CTC from patient COL 05. Furthermore, CTCs from patient COL 07 showed mutational heterogeneity: only two of them presented the same variants in APC and KRAS genes: both CTCs showed the APC p.Q1367* mutation in homozygosis, while the KRAS p.G12S mutation was in heterozygosis in CTC1 and in homozygosis in CTC2. The APC p.Q1367* mutation was detected also in cfDNA from the same patient.

#### cfDNA sequencing

Cell-free DNA from 20 basal blood draws from KRAS mutated mCRC patients was sequenced by NGS for a panel of CRC related genes as reported in the ‘Methods’ section. Sequencing data are reported in Supplementary Table S3. A median quantity of 9.5 ng cfDNA (range 2.8–20) was used to prepare libraries with a final median concentration of 183 pM (range 82–536 pM). The median read coverage was 33,264 (range 4776–128,852).

#### Comparison of the mutational status of KRAS between cfDNA and FFPE tumour tissue

The mutational status of KRAS gene was compared between FFPE tumour tissues and basal cfDNA samples from the same patient. The results are reported in Table 3. The length of time between the diagnosis, with the assessment of FFPE tissue mutational status, and cfDNA basal blood draw at patients’ enrolment ranged from 0 to 33 months. For one sample we did not reach a sufficient coverage to assess the mutational status of the KRAS hot spot regions of interest. 17 out of 19 samples showed at least one mutation in KRAS with 89.5% concordance with the mutational status of the FFPE tumour tissues. The concordance of the mutational status of tissue and cfDNA seems to be independent from the duration of the time interval between diagnosis (time of biopsy) and enrolment (time of blood draw).

**Table 3:** Comparison of KRAS mutational status in FFPE tumoral tissues and cfDNA.

	Tumour tissue	Plasma	
pt id	FFPE TISSUE KRAS mutations	cfDNA KRAS mutations	cfDNA KRAS allele frequency
COL 01	KRAS p.G12D	KRAS p.G12D	0.83
COL 02	KRAS p.G12C	KRAS p.G12C	65.26
COL 03	KRAS p.G12A	KRAS p.G12A	7.25
COL 04	KRAS p.G12D	KRAS p.G12D	30.33
COL 05	KRAS p.G13D	KRAS p.G13D	13.68
COL 06	KRAS p.G12C	KRAS p.G12C	1.92
COL 07	KRAS p.G12S	KRAS p.G12S	38.19
COL 08	KRAS p.G12D	KRAS p.G12D	1.36
COL 09	KRAS p.G13D	KRAS p.G13D	28.78
COL 10	KRAS p.G13D	KRAS p.G13D	2.40
COL 11	KRAS p.G12D	KRAS p.G12D	2.78
COL 12	KRAS p.G12A	KRAS p.G12A	38.00
COL 13	KRAS p.G13D	KRAS p.G13D	0.38
		KRAS p.G12D	1.88
COL 14	KRAS p.G12A	KRAS p.G12D	1.83
COL 15	KRAS p.G13V	WT	
COL 16	KRAS p.G12D	KRAS p.G12D	0.56
COL 17	KRAS p.A146K	WT	
COL 18	KRAS p.G12D	KRAS p.G12D	21.81
COL 19	KRAS p.G12C	KRAS p.G12C	10.75
COL 20	KRAS p.G12D	nd	nd

*FFPE*  formalin fixed paraffin emebedded, *nd*  not determined.

Two cases did not show any KRAS mutation in plasma while the tissue was KRAS mutated. In these two cases an interval of 7 and 5 months, respectively, occurred between diagnosis and enrolment.

Focusing on the specific mutations detected in FFPE tissue samples, a perfect concordance was evidenced for 16/19 samples (84%), while one of them presented two different variants in FFPE and cfDNA on the same codon (KRAS p.G12A and KRAS p.G12D, respectively).

The mutational status of KRAS gene in cfDNA was concordant with that of 2/4 single CTCs from patient COL 07 presenting the mutation KRAS p.G12S, but not with the single CTC from patient COL 05, wild type for KRAS codons 12 and 13.

#### Sequence variants detected in other genes from the panel

Nineteen cfDNA samples presented sequence variants in other genes of the tested panel (Table 4) for a total of 49 somatic hot spot mutations detected in cfDNA, with a median number of 2 per patient (range 1–5).

**Table 4:** Mutational status of the other genes of the panel on cfDNA.

Sample name	Gene ID	Variant	Type	Frequency %
cfDNA COL 01	FBXW7	p.R465C	SNV	0.55
cfDNA COL 02	APC	p.S1465fs	DEL	39.75
	SMAD4	p.R361H	SNV	57.33
cfDNA COL 03	TP53	p.G154D	SNV	0.57
cfDNA COL 04	FBXW7	p.R465C	SNV	30.45
	GNAS	p.R201H	SNV	0.64
cfDNA COL 06	APC	p.Q1367*	SNV	0.33
		p.A1492fs	INDEL	1.20
	FBXW7	p.R465C	SNV	1.14
	MAP2K1	p.E203K	SNV	0.16
	TP53	p.H179R	SNV	1.73
cfDNA COL 07	APC	p.Q1367*	SNV	76.21
	TP53	p.C176Y	SNV	0.07
cfDNA COL 08	APC	p.S1371D	MNV	0.41
	FBXW7	p.R465C	SNV	3.33
	TP53	p.G245D	SNV	0.55
cfDNA COL 09	APC	p.R876*	SNV	28.74
	TP53	p.E286K	SNV	0.16
		p.R248W	SNV	20.74
		p.R213Q	SNV	0.11
		p.R175H	SNV	0.32
cfDNA COL 10	PIK3CA	p.E542K	SNV	0.58
	TP53	p.R175H	SNV	1.40
cfDNA COL 11	APC	p.Q1367*	SNV	0.28
	FBXW7	p.R465C	SNV	1.24
	TP53	p.H214fs	INDEL	0.46
cfDNA COL 12	TP53	p.R213Q	SNV	0.14
		p.R248Q	SNV	60.11
cfDNA COL 13	APC	p.R876*	SNV	0.25
	FBXW7	p.R465C	SNV	2.14
	TP53	p.H214fs	INDEL	0.27
		p.Y220T	MNV	0.96
cfDNA COL 14	FBXW7	p.R465C	SNV	1.72
	SMAD4	p.R361H	SNV	0.25
cfDNA COL 15	APC	p.E1464fs	INDEL	5.26
	PIK3CA	p.E542K	SNV	5.17
	TP53	p.R248W	SNV	3.89
cfDNA COL 16	FBXW7	p.R465H	SNV	0.06
	TP53	p.C141Y	SNV	0.09
		p.P278L	SNV	0.82
cfDNA COL 17	APC	p.S1465fs	INDEL	0.07
	TP53	p.Y220C	SNV	0.07
		p.R248W	SNV	0.11
		p.R273H	SNV	40.46
cfDNA COL 18	APC	p.E1464fs	INDEL	0.13
	FBXW7	p.R465C	SNV	0.19
	TP53	p.G266*	SNV	25.99
cfDNA COL 19	PIK3CA	p.E545K	SNV	1.48
cfDNA COL 20	TP53	p.G244D	SNV	0.10

The most frequently mutated gene was TP53, followed by APC, FBXW7, PIK3CA and SMAD4.

In particular:

- 14/20 samples (70%) had different variants in TP53;
- 8 samples showed the same FBXW7 variant (p.R465C), which is frequently described in CRC. Another sample presented a different variant in the same codon, for a total of 9/20 (45%) patients showing FBXW7 mutations in cfDNA;
- 3/20 samples presented the same APC mutation (p.Q1367*) and other 7 plasma samples showed mutations in the same gene which has a key role in CRC tumorigenesis;
- 3/20 (15%) samples showed mutations in PIK3CA, two of them sharing the p.E542K mutation;
- 2/20 (10%) samples shared the SMAD4 p.R361H mutation, frequently detected in CRC. Both patients with this SMAD4 mutation showed a significantly different OS with respect to the other patients (*p* = 0.02) according to Kaplan–Meier estimates.

### Longitudinal monitoring by liquid biopsy data

**Fig. 2:**
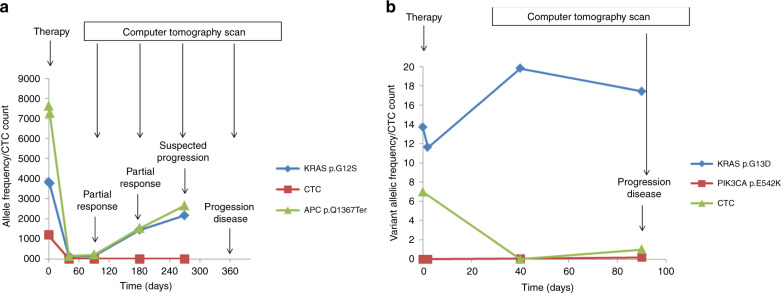
Longitudinal disease monitoring in two mCRC patients by liquid biopsy evaluation. In the first subject (**a**) CTCs disappear from the circulation at 40 days while two mutations in KRAS and APC detected in cfDNA indicate an increase of tumour cfDNA during treatment. In the timeline, therapy and in vivo analysis test schedule is also reported for a better framing of results. Tumour cfDNA seems to anticipate conventional diagnostic tools in predicting PD. In the second patient (**b**) the appearance of one CTC at 90 days corresponds to progression of the disease (PD).

Here we report the graphical representation of the results of the longitudinal monitoring by liquid biopsy from two patients of our study cohort (Fig. 2). In the first case (Fig. 2a), a 53-year-old female came to our attention for a CRC with synchronous multiple lung and hepatic metastases. After primary tumour surgery, the patient received 12 cycles of first-line CT with FOLFOXIRI/bevacizumab, followed by maintenance de Gramont bevacizumab, obtaining partial response at 3 and 6 months of treatment. Simultaneously, CTC count and APC (p.Q1367*) and KRAS (p.G12S) mutations in cfDNA disappeared from the circulation at 40 days. After 6 months of treatment, APC and KRAS mutations in cfDNA progressively increased, anticipating the radiological PD occurred after a total of 12 months of treatment. The patient received FOLFIRI bevacizumab as second-line treatment for 3 months without a response and died 3 months after the treatment was discontinued.

In the second case (Fig. 2b), a 60-year-old male came to our attention for a CRC with synchronous multiple hepatic and abdominal lymph node metastases. He received a first-line CT with FOLFOX bevacizumab. After 6 cycles, however, a CT scan showed PD. Seven CTCs were identified at baseline; then, after an initial decrease in CTC count (no cell detected at day 40), one CTC was detected at day 90, concurrently with the radiological PD, while ctDNA levels (KRAS p.G13D mutation in cfDNA) varied over time. The patient received second-line chemotherapy with FOLFIRI aflibercept for 6 cycles until the disease invariably progressed and the patient died 11 months after the initial diagnosis.

## Discussion

Despite the advancement in the management of patients with mCRC, some issues related to diagnosis, prognosis and follow up are still to be tackled.^6^

The study of the liquid biopsy has opened the perspective of a non-invasive tumour diagnosis and characterisation and it has been applied to the early detection of cancer, identification of therapeutic targets and monitoring the response to treatments, as well as the disclosure of mechanisms underlying disease progression.^2^ However, the analysis of the liquid biopsy is a difficult and multi-step process and relies on a plethora of methods whose standardisation cannot be accomplished without considering all the technical challenges related to the entire workflow, including the pre-analytical phase.

The whole experimental design for liquid biopsy analysis allowed the achievement of data related to CTC counting and imaging and to their molecular characterisation together with the analysis of cfDNA starting from 20 ml blood for each patient. Overall, we demonstrated the feasibility of cell-free DNA and CTC characterisation in our cohort of patients: at baseline CTC enumeration was successful in 90% of the patient population (18 out of 20 patients), while single-cell analysis was only feasible for 2 out of 20 patients due to the rarity of CTC detection and the low number of CTCs identified; NGS sequencing of cfDNA reached a sufficient coverage for the analysis of the complete panel of genes of interest in 95% of the patient population (19 out of 20 patients).

The percentage of CRC patients with more than 3 CTCs/7.5 ml of blood at basal blood draw was consistent, albeit slightly lower, with that reported in the literature^6–8^ for samples not selected for the presence of KRAS mutations processed with the same method. Although the FDA-approved application of the CellSearch® system to metastatic colon cancer patients implies a cut-off of 3 CTCs/7.5 ml blood, we preferred to stratify the patients of our cohort as negative or positive for the presence of CTCs, since studies on CTCs in colorectal cancer patients using the CellSearch® system showed lower CTC numbers than in metastatic breast and prostate cancer patients (the other tumours for which the CellSearch® is FDA-approved^9^ and it has been demonstrated that the presence of even a single CTC in the peripheral blood of patients with mCRC significantly affects their prognosis.^10^ The presence of CTCs at basal blood draw before treatment was confirmed to be a prognostic marker, as assessed by Kaplan–Meier estimates of probabilities of OS, moreover, all the patients with a PFS of at least 13 months did not present any CTC at baseline. Considering the best response to first-line CT the percentage of CTC positive patients was higher in those who had PD as best response than in those with SD or PR.

Single CTC analysis, although feasible in a limited number of samples, confirmed previous findings on CTC heterogeneity^5,11,12^ and showed some correlation with the mutational status of cfDNA and tissue from the same subject. The combination of CellSearch® with diagnostic leukapheresis has been proposed as a solution to improve the detection rate and the number of CTCs,^13^ but this could be a stressful procedure for the patient, especially in the metastatic setting.

The results of comparison between CTCs and cfDNA underline the importance of using both samples to acquire more comprehensive information about the disease. Moreover, the mutational analysis of single CTCs may help in identifying subpopulations of cancer cells responsible for metastasis that may require different treatments.^14^

The percentage of cfDNA samples with mutations in the most common CRC driver genes, such as APC, KRAS, PIK3CA, SMAD4 and TP53, was concordant or comparable with what reported in the literature for CRC tissues. In particular, TP53, a key driver gene^15^ in CRC with a prevalence of mutations of 50-75%^16^ was mutated in 70% of our samples. The percentage of cfDNA samples (50%) with mutations in APC, which has a key role in CRC tumorigenesis,^15^ was comparable with what reported for CRC tissues (>60% ref. ^16^). The percentage of patients’ samples showing mutations in PIK3CA was concordant with that already reported for CRC (10–15% ref. ^17^). Analogously, the prevalence of SMAD4 mutations in our cohort of patients was the same as that already reported for CRC (10%).^18^ This gene is a major contributor to CRC tumorigenesis and mutations in this gene are considered an adverse prognostic marker, as confirmed by our data showing a significantly shorter OS in two patients bearing the SMAD4 p.R361H mutation.

On the contrary, the frequency of mutations in the tumour suppressor gene FBXW7, usually associated to a negative prognosis,^19^ in our cohort of patients was much higher than what reported in the literature (10% prevalence^20^). This could be related to the characteristics of patients who have been selected on the basis of the presence of KRAS mutations. In fact, it has been observed that FBXW7 mutations in advanced cancers are rarely isolated and frequently occur in concomitance with KRAS mutations especially in advanced CRC.^21^

Considering the percentage of cfDNA samples with mutations in CRC driver genes reported in the literature,^6^ our results were within the range for APC, KRAS, PIK3CA and TP53. We did not find any sequence variant in BRAF or NRAS genes, reported to be mutated in cfDNA from CRC patients at a frequency ranging from 5 to 10% and from 3 to 8%, respectively.^6^ The great variability of driver genes mutation rates in cfDNA reported in previous studies may be due to the different pre-analytical and analytical settings affecting the results.

Some authors investigated the concordance between cfDNA and corresponding tissues for KRAS mutational status in CRC patients, finding highly variable results ranging from 14% concordance to 100%.^6^ In our cohort of patients, we found an optimal concordance (89.5% for the presence of any mutation in KRAS, 84% for the specific KRAS mutation), independently from time interval between tissue and cfDNA sequencing, which in some cases reached 3 years.

Single CTC mutational status in one patient was heterogeneous and correlated with that of cfDNA and tissue.

Two representative examples of longitudinal monitoring by liquid biopsy showed that in one case tumour cfDNA anticipated conventional diagnostic tools in predicting PD, while in the other one the appearance of one CTC at 90 days corresponded to PD.

Our results confirm that CTCs and cfDNA represent two complementary aspects of the liquid biopsy both worth investigating; in fact, both seem to exert in our study a potential prognostic role, which requires confirmation in larger studies, due to the limited sample size of our cohort.

Moreover, the real strength of the liquid biopsy emerges from the longitudinal study of the patients with the opportunity to obtain, with minimal stress for the patient, information merging with the clinical evaluation of the disease development.

## Supplementary information

SUPPLEMENTARY DATA

## Data Availability

The datasets analysed during the current study are available from the corresponding author on reasonable request.
