# Supplementary material for: Circulating tumour cells and cell-free DNA as a prognostic factor in metastatic colorectal cancer: the OMITERC prospective study
Source: Br J Cancer. 2021 May 5;125(1):94–100. doi: 10.1038/s41416-021-01399-6 (PMC8257609; doi:10.1038/s41416-021-01399-6)
Supplement: Supplementary file 1 — SUPPLEMENTARY DATA [file 41416_2021_1399_MOESM1_ESM.docx]

Supplementary Table S1A. CRC patients’ clinical characteristics.

| **Characteristics** | **Patients (n = 20)** |
| --- | --- |
| *Sex* | |
| Male | 10 (50%) |
| Female | 10 (50%) |
| *Age (years)* | |
| Median | 65 (45 – 84) |
| <70 | 13 (65%) |
| ≥70 | 7 (35%). |
| *ECOG PS* | |
| 0 | 16 (80%) |
| 1-2 | 4 (20%) |
| *Primary tumor side* | |
| Left | 8 (40%) |
| Right | 12 (60%) |
| *Resection of primary tumor* | |
| Yes | 12 (60%) |
| No | 8 (40%) |
| *Colloid carcinoma* | |
| Yes | 5 (5%) |
| No | 15 (95%) |
| *Grading* | |
| G1 | 0 (0%) |
| G2 | 12 (60%) |
| G3 | 1 (5%) |
| Not assessed | 7 (35.0%) |
| *Number of metastatic sites* | |
| 1 | 6 (30%) |
| 2 | 7 (35%) |
| ≥3 | 7 (35%) |

*Note. ECOG (Eastern Cooperative Oncology Group) performance status (PS).*

Supplementary Table S1B. Sites of metastases in CRC patients of our cohort.

| Patient ID | liver | lung | lymph nodes* | ascites | pleural effusion | other sites |
| --- | --- | --- | --- | --- | --- | --- |
| COL 01 | x | - | x | x | x | x |
| COL 02 | x | x | x | x | - | x |
| COL 03 | x | x | x | - | - | - |
| COL 04 | x | - | - | - | - | - |
| COL 05 | x | - | x | - | - | - |
| COL 06 | - | x | x | - | - | - |
| COL 07 | x | x | x | - | - | x |
| COL 08 | - | - | x | x | - | x |
| COL 09 | x | - | x | - | - | - |
| COL 10 | - | - | x | - | - | x |
| COL 11 | x | - | - | - | - | - |
| COL 12 | x | - | - | - | - | - |
| COL 13 | x | x | x | - | - | x |
| COL 14 | - | - | x | - | - | x |
| COL 15 | x | - | - | - | - | - |
| COL 16 | - | x | - | - | - | x |
| COL 17 | x | - | - | - | - | - |
| COL 18 | x | x | - | - | - | - |
| COL 19 | x | x | x | - | - | - |
| COL 20 | x | - | - | - | - | - |

*Note. *= abdominal or supradiaphragmatic.*

Supplementary Table S2. Ion AmpliSeq Cancer Hotspot Panel v2 target genes.

| ABL1 | EGFR | GNAS | KRAS | PTPN11 |
| --- | --- | --- | --- | --- |
| AKT1 | ERBB2 | GNAQ | MET | RB1 |
| ALK | ERBB4 | HNF1A | MLH1 | RET |
| APC | EZH2 | HRAS | MPL | SMAD4 |
| ATM | FBXW7 | IDH1 | NOTCH1 | SMARCB1 |
| BRAF | FGFR1 | JAK2 | NPM1 | SMO |
| CDH1 | FGFR2 | JAK3 | NRAS | SRC |
| CDKN2A | FGFR3 | IDH2 | PDGFRA | STK11 |
| CSF1R | FLT3 | KDR | PIK3CA | TP53 |
| CTNNB1 | GNA11 | KIT | PTEN | VHL |


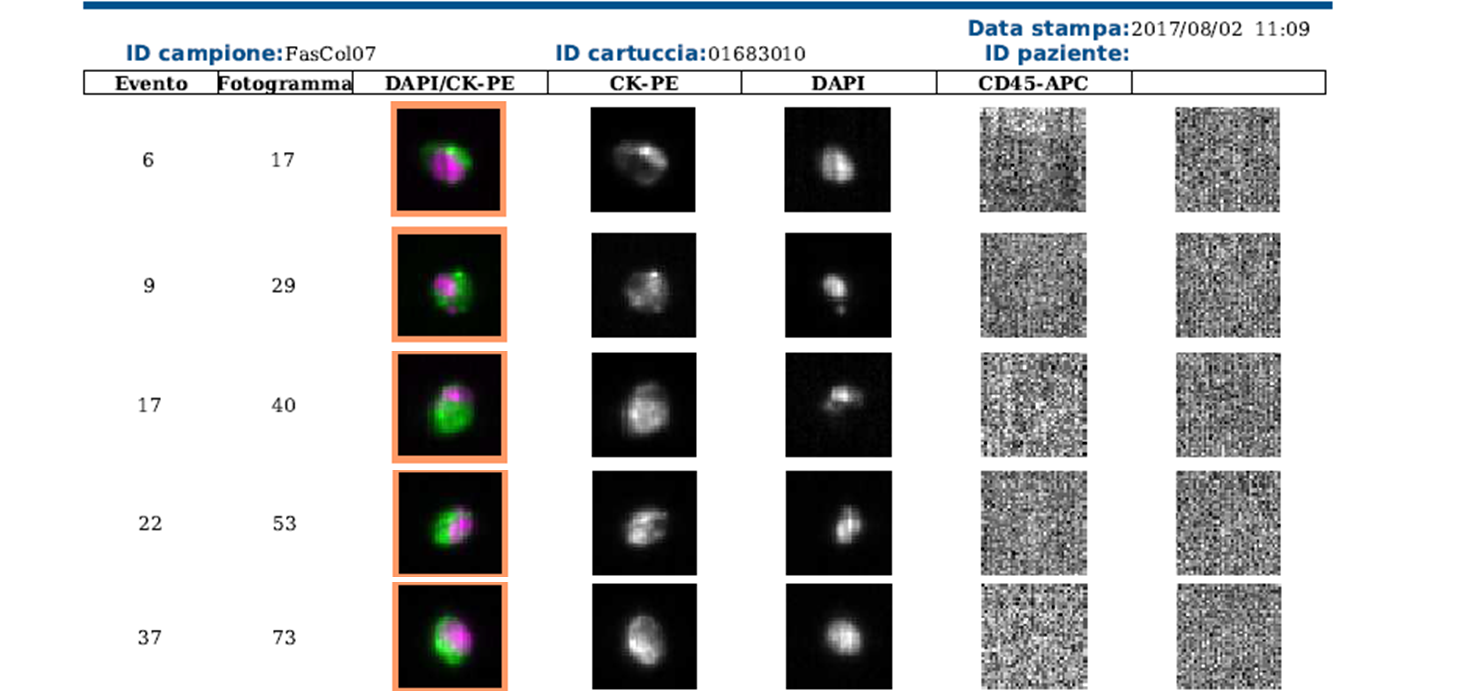


Supplementary Figure S1. Examples of representative CTCs detected in mCRC patients as they appear in the CellSearch® image gallery. CK-PE: fluorescent signals related to anti-cytokeratin 8, 18 and 19-(PE labelled antibodies); CD45-APC: fluorescent signals related to anti-CD45 antibody labelled with APC; DAPI: fluorescent signals related to DNA intercalating dye.

Supplementary Table S3. Sequencing parameters for basal cfDNA samples.

| sample | input cfDNA (ng) | library yield (pM) | mean depth | n° mapped reads | % reads on target | Median Read Coverage | Median Mol Coverage |
| --- | --- | --- | --- | --- | --- | --- | --- |
| cfDNA COL 01 | 7,8 | 187 | 46672 | 2317676 | 92,83% | 38158 | 1812 |
| cfDNA COL 02 | 20 | 466 | 49438 | 2679850 | 90,43% | 43282 | 3322 |
| cfDNA COL 03 | 7,3 | 170 | 30957 | 1725010 | 87,94% | 26295 | 1161 |
| cfDNA COL 04 | 20 | 522 | 50972 | 2671334 | 91,67% | 43894 | 3652 |
| cfDNA COL 05 | 16,8 | 141 | 43511 | 2103184 | 93,94% | 36539 | 2967 |
| cfDNA COL 06 | 6,1 | 102 | 52412 | 2695541 | 92,14% | 43205 | 1117 |
| cfDNA COL 07 | 20 | 530 | 40594 | 2179362 | 90,62% | 33027 | 3326 |
| cfDNA COL 08 | 3,38 | 82 | 43289 | 2342075 | 87,04% | 33501 | 684 |
| cfDNA COL 09 | 20 | 422 | 48665 | 2515014 | 91,93% | 39945 | 3546 |
| cfDNA COL 10 | 2,8 | 236 | 5688 | 535833 | 80,13% | 4776 | 346 |
| cfDNA COL 11 | 4,9 | 107 | 34625 | 1839252 | 88,95% | 28149 | 955 |
| cfDNA COL 12 | 20 | 178 | 84120 | 5293756 | 83,76% | 72110 | 2094 |
| cfDNA COL 13 | 3,5 | 111 | 31419 | 1803673 | 85,99% | 26509 | 648 |
| cfDNA COL 14 | 5,5 | 129 | 33297 | 1926603 | 86,39% | 28004 | 822 |
| cfDNA COL 15 | 13,9 | 131 | 132564 | 7129375 | 93,81% | 128852 | 2809 |
| cfDNA COL 16 | 20 | 155 | 109225 | 5915192 | 90,46% | 95524 | 3167 |
| cfDNA COL 17 | 20 | 474 | 30611 | 1971589 | 88,46% | 27336 | 1943 |
| cfDNA COL 18 | 3,6 | 261 | 16453 | 1267984 | 82,35% | 14585 | 886 |
| cfDNA COL 19 | 6,5 | 536 | 19825 | 1617780 | 81,03% | 18207 | 1136 |
| cfDNA COL 20 | 11,1 | 310 | 30285 | 1549137 | 94,84% | 26199 | 1739 |
| Median  (range) | 9,5  (2,8-20) | 183  (82-536) | 41942  (5688-132564) | 2141273  (535833-7129375) | 89,69%  (80,13-94,84) | 33264  (4776-128852) | 1776  (346-3652) |
